# Resting segmental speckle tracking strain and strain rate in stable coronary artery disease and revascularized myocardial infarction

**DOI:** 10.1007/s10554-024-03200-0

**Published:** 2024-08-23

**Authors:** Hatice Akay Caglayan, Didrik Kjønås, Mikhail Kornev, Amjid Iqbal, Mehran Jazbani, Assami Rösner

**Affiliations:** 1 Department of Cardiology, Division of Cardiothoracic and Respiratory Medicine, University Hospital of North Norway, Tromsø, Norway; 2 Department of Clinical Medicine, UiT The Arctic University of Norway, Tromsø, Norway; 3 Department of Gastrointestinal surgery, University Hospital of North Norway, Tromsø, Norway; 4 Department of Cardiology, Oslo University Hospital, Rikshospitalet, Oslo Norway; 5 Department of Cardiology, Stavanger University Hospital, Stavanger, Norway

**Keywords:** Coronary artery disease, Myocardial infarction, Speckle tracking imaging, Segmental strain and strain rate, Peak early diastolic strain rate

## Abstract

Patients with acute coronary artery disease (CAD) exhibit reduced global and regional strain and strain rate (S/SR). However, knowledge about segmental S/SR in stable CAD patients is still limited. This study aimed to investigate whether resting segmental S/SR measurements differ in patients with chronic chest pain who have normal coronary arteries or stenotic coronary arteries, and to compare these measurements to those in patients with revascularized myocardial infarction (MI). We prospectively enrolled 510 patients with chronic chest pain referred for coronary computed tomography angiography (CCTA) and 102 patients revascularized after MI. All participants underwent transthoracic echocardiography featuring S/SR analysis. In addition to the patients with MI, patients with suspected CAD based on CCTA findings subsequently underwent invasive coronary angiography (CAG). We assessed global longitudinal strain (GLS) and averaged segmental peak longitudinal strain during systole (PLS), peak systolic strain rate (SRs), peak early diastolic strain rate (SRe), and post systolic shortening (PSS). We also determined functionally reduced segment percentages using differing S/SR cut-off values. There were significant disparities in all average segmental S/SR metrics between the No-CAD and MI groups. SRe was the only S/SR metric that differed significantly between the No-CAD and PCI groups. Differences in SRe, PLS and GLS measurements were observed between the No-CAD and CABG groups. The proportion of diminished segmental S/SR mirrored these findings. For the percentage of pathological segments with varying cut-off values, segmental SRe below 1.5 s^− 1^ displayed the most marked difference among the four groups (*p* < 0.001). Revascularized MI patients or those referred to CABG present with diminished segmental S/SR values. However, among patients with chronic chest pain, only segmental SRe discerns subtle disparities between the No-CAD and the PCI group. The diagnostic accuracy of SRe warrants further exploration in subsequent studies.

**Supplementary Information:**

The online version contains supplementary material available at 10.1007/s10554-024-03200-0.

## Introduction

Coronary artery disease (CAD) is a leading cause of morbidity and mortality worldwide [1]. Early and accurate diagnosis of CAD is crucial for preventing complications like myocardial infarction (MI), heart failure (HF) and death [1]. In patients with chronic chest pain and suspected CAD, non-invasive functional or anatomical tests are recommended as initial diagnostic tools [1]. However, functional non-invasive tests such as stress echocardiography have limitations of being time-consuming, costly, and having low sensitivity for detecting mild CAD [1, 2]. Coronary computed tomography angiography (CCTA) allows anatomical assessment with high sensitivity for CAD stenosis but has downsides of radiation, cost, and limited accessibility [1–3].

Thus, an accessible, non-invasive test to diagnose CAD in the vast population of chest-pain patients is an unmet need. Stable CAD causes repetitive ischemia in myocardial segments supplied by the affected coronary artery (CA) leading to impaired regional function during stress. Strain/strain rate (S/SR) imaging has recently been used to identify subtle myocardial dysfunction and early diseases [4, 5]. A recent study applying S/SR imaging demonstrated that these changes can also be shown at resting echocardiography in patients with stable CAD [6].

Prior studies have shown that global longitudinal strain (GLS) and regional longitudinal strain (RLS) are reduced in stable CAD [7–10]. Early diastolic strain rate (SRe) and peak systolic strain rate (SRs) were also reduced in stable CAD [4, 11, 12]. However, knowledge about segmental S/SR patterns in stable CAD remains limited. Our research group has previously demonstrated that the presence of strain curve artefacts results in reduced segmental S/SR values in the assessment of myocardial function [13]. Previous studies have reported that the majority of patients with acute MI within 1–13 days after revascularization continue to display characteristic patterns of reduced regional myocardial function specifically related to the affected vascular territory [14–16]. Thus, to accomplish assessment of myocardium in the presence of artefacts, hearts affected by stunning after an acute MI appear to be the most suitable reference for evaluating regional function changes in CAD.

This study aimed to:

1. Investigate differences in resting segmental S/SR between patients with non-stenotic versus stenotic CA among individuals with chronic chest pain.
2. Compare segmental S/SR in these groups to patterns in revascularized MI to account for the effect of artefacts on segmental analyses at the presence of pathology.

We hypothesized that detailed segmental S/SR analysis may improve diagnosis of CAD compared to global strain analyses. Investigating segmental S/SR differences between patients with chronic chest pain who had stenotic coronary arteries and MI patients as a reference can provide insights into subtle myocardial dysfunction in stable CAD.

## Methods

### Study population

Our study is a prospective observational study conducted between 2016 and 2021. We included 510 patients with chronic chest pain (the chest pain group) and 102 patients with acute MI (the MI group) from the Cardiology Department at the University Hospital of North Norway, Tromsø. In the chest pain group, patients referred for CCTA were preselected by a cardiologist based on their chest pain history, exercise electrocardiogram (ECG), family history, and risk factors. Exclusion criteria for this study were atrial fibrillation (AF), left bundle branch block (LBBB), previously known CAD, or any structural heart disease. These criteria were implemented to minimise the impact of these specific heart conditions on myocardial function, as these conditions can significantly affect myocardial function and might lead to misinterpretations of the study results. For the MI group, we included patients with acute ST-elevation MI or non-ST-elevation MI within 1–3 days after revascularization. Exclusion criteria were; history of previous MI, three-vessel disease, and referral to coronary artery bypass graft (CABG). Based on CCTA and coronary angiography (CAG) results, patients in the chest pain group were divided into the No-CAD group (defined as no or no treatment-relevant CA-stenosis), the percutaneous coronary intervention (PCI) group, the CABG group, and compared with the MI group.

### Patient demographics and clinical characteristics

All included patients filled out a questionnaire regarding their symptoms and risk factors. We obtained clinical data, lipid profile, creatinine, Hba1c, and fasting glucose levels from the patients` electronic medical records. Before the echocardiographic examination, blood pressure, height, weight, body mass index (BMI) were measured and a 12-lead ECG was conducted. Use of prescribed medicines for the chest pain and MI groups was recorded at the time of admission to the hospital. Patients with a medical history of diabetes mellitus (DM) -the use of medication for DM, or fasting glucose level > 126 mg/dl (7.0 mmol/L), or Hba1c > 6.5% (48 mmol/mol) were defined as having DM. Patients with a medical history of hypertension (HT) -and drug use for HT or blood pressure > 140/90 were defined as having HT. Patients with a medical history of chronic obstructive pulmonary disease (COPD) and use of medication or an obstructive pattern consistent with COPD of spirometry were defined as having COPD.

### Echocardiography, strain and strain rate analysis

All included patients with chest pain underwent echocardiography the same day as CCTA. CAG was performed 1–4 weeks after CCTA if indicated. MI patients underwent echocardiography 1–3 days after CAG and PCI. Echocardiographic examination was performed in the left lateral decubital position using Vivid E9 or E95 ultrasound machines (GE, Horton, Norway) with a 5 − 1 MHz transducer. Conventional 2-dimensional (2D) greyscale images were obtained from parasternal long and short axis views and apical two, three and four chamber views. Pulsed Doppler flow across the mitral valve -and left ventricular outflow tract (LVOT), and continuous and colour Doppler across the aortic valve were obtained from the apical four and five chamber views. Peak R of the QRS was used as the beginning of the cardiac cycle to avoid underestimating segmental peak-strain values. Three consecutive cardiac cycles of apical 2D greyscale images were recorded at rest in apical two-, three- and four-chamber views. Frame rates were set above 45 frames per second (Fps) for the 2D grayscale images. Images were stored digitally in a cine-loop format for strain analyses.

The strain analyses of 2D images were performed offline using Q-analyses function of EchoPac (GE, Horton, Norway). Pulsed wave Doppler of the LVOT was used to measure the aortic valve opening and closing times. Aortic valve closure was defined as end-systole. Subsequently, the endocardial border was traced manually in end-systole. Myocardial longitudinal curves were used for strain analyses. Numerical and graphical S/SR were automatically calculated for all six segments from each view and stored by EchoPac software program. Visual assessment of screenshots of strain curves were used to identify and discard artefactual strain curves from segmental analysis. To determine the percentage of pathological segments, we defined five graded cut-off values for each S/SR parameter, ranging from lower normal to highly abnormal. The chosen cut-off values for SRe were: 2.5 s^− 1^, 2.0 s^− 1^, 1.5 s^− 1^, 1.0 s^− 1^, 0.75 s^− 1^. The cut-off values for SRs were − 1.4 s^− 1^, -1.2 s^− 1^, -1,0 s^− 1^, -0.8 s^− 1^, and − 0.6 s^− 1^. For PLS the cut-off values were − 16%, -14%, -12%, -10%, and − 8%. For post systolic shortening (PSS) we chose cut-off values at -5%, -4%, -3%, -2%, and − 1%.

We defined a myocardial segmental function as low if its measured SRe, SRs, PLS or PSS value was below the defined cut-off. We determined the percentage of segments with reduced function for each patient by dividing the number of segments with values below each cut-off by the total number of segments analyzed. We then compared the mean percentage of pathological segments at each graded cut-off value between the No-CAD, PCI, CABG and MI groups.

### Invasive coronary angiography

All patients with positive or inconclusive CCTA results and all MI patients underwent CAG. CAG was performed by the percutaneous radial or femoral approach. Multiple views of each vessel were derived. A reduction > 70% in the arterial lumen for the right coronary artery, left anterior descending artery, circumflex artery, and a 50% reduction in the arterial lumen for the left main coronary artery were considered significant. Patients who had significant stenosis received revascularization treatment at the same time. Patients with CABG indications were directed to surgical operation based on the angiographic findings.

### Statistics

Statistical analysis was performed using SPSS software versions 27 and 28 (SPSS Inc., Chicago, IL). Continuous variables were expressed as mean ± standard deviation (SD), while categorical data were expressed as absolute numbers of percentages. Baseline characteristics of the No-CAD, PCI, CABG and MI groups were compared using one-way analysis of variance (ANOVA) with Bonferroni post-hoc test for continuous variables and Pearson’s chi-squared test for categorical variables. Comparison of S/SR measurements and percentage of pathological segments for five different cut-off values of PLS, SRe, SRs, and PSS among the groups was performed using ANOVA with Bonferroni post-hoc test. A *p*-value of ≤ 0.05 level was considered statistically significant.

### Reproducibility

To assess intra-observer variability, the investigator who performed the initial analysis re-analysed the data obtained from randomly selected 25 patients 6–12 months after the first analysis. For assessment of inter-observer variability, the data obtained from randomly selected 25 patients were analysed by a second investigator, who was blinded to the results of the previous investigator. Bland-Altman plots were used to assess intra- and inter-observer agreement.

## Results

A total of 510 patients with chronic chest pain and 102 patients with acute MI were included in this study. Among these 510 chest pain patients, seven patients had LBBB, two had AF, one had cardiomyopathy, two had pericardial disease, one had an atrial septal defect, and one had poor image quality; thus a total of 13 patients were excluded. Finally, 496 patients with chronic chest pain were included in the study. Among 496 patients, 190 patients with positive or inconclusive CCTA subsequently underwent CAG. Among patients who had CAG examination, 94 patients underwent PCI, eight patients underwent CABG, and three patients had significant stenosis but did not receive PCI or CABG treatments.

We summarized the baseline demographic and clinical characteristics of the four patient groups in Table 1. A total of 598 patients were analysed; their mean age was 60.4, and 59.5% of the patients were male. Based on CCTA and CAG results, the patients were divided into four groups. Group 1 had 394 patients with No-CAD (the No-CAD group), including 51.3% males; Group 2 had 94 patients who received PCI intervention (the PCI group), 74.5% male; Group 3 had 8 patients who received CABG intervention (the CABG group), 75% male; and Group 4 had 102 patients who had MI (the MI group), 76.5% male. The patients in the PCI and MI groups were older and more frequently male compared to the those in the No-CAD and CABG groups. The patients in the CABG and MI groups had higher BMI and smoking levels. The MI group had lower systolic and diastolic blood pressure and a lower prevalence of use of statins. However, the prevalence of the use of angiotensin-converting enzyme inhibitors (ACEIs)/ angiotensin II receptor antagonists (ARBs) was higher in the MI group. More patients with DM and HT were observed in the MI group than in the No-CAD group. There were no statistically significant differences between groups in terms of HT, the prevalence of COPD, the use of beta-adrenergic blockers (BB), creatinine and cholesterol levels.

**Table 1:** Clinical characteristics of the study population

	No-CAD	PCI	CABG	MI	All patients	*P*-value
	N (%) or mean ± SD	N (%) or mean ± SD	N (%) or mean ± SD	N (%) or mean ± SD	N (%) or mean ± SD	
N	394	94	8	102	598	
Male	202(51.3%)	70(74.5%)*	6(75.0%)	78(76.5%)*	356 (59.5%)	< 0.001
Age (y)	58.6 ± 10.8	62.8 ± 9.0*	61.2 ± 10.4	65.5 ± 11.1*	60.4 ± 10.9	< 0.001
BMI	26.45 ± 3.6	27.16 ± 3.06	31.03 ± 4.2*†	28.11 ± 3.5*	26.8 ± 3.5	0.002
Smoking	69(17.5%)	22(23.4%)	3(37.5%)*†	36(35.3%)*†	130(21.7%)	< 0.001
Diabetes	24(6.1%)	12(12.8%)	1(12.5%)	18(17.6%)*	55(9.2%)	0.002
Hypertension	117(29.8%)	39(41.5%)	3(37.5%)	50(49.0%)*	209(35.1%)	0.002
COPD	24(6.1%)	9(9.6%)	1(12.5%)	8(7.8%)	42(7.0%)	0.597
Cholesterol (mmol/L)	5.2 ± 1.2	5.1 ± 1.2	5.5 ± 1.6	5.2 ± 1.0	5.1 ± 1.2	0.638
Creatinine (µmol/L)	73.7 ± 14.6	78.5 ± 18.5	76.5 ± 11.6	78.0 ± 21.0	75.3 ± 16.6	0.025
Systolic blood pressure (mm Hg)	137.9 ± 17.8	143.0 ± 20.0	155.0 ± 22.1	133.6 ± 24.1†‡	138.2 ± 19.7	< 0.001
Diastolic blood pressure (mm Hg)	86.8 ± 10.9	86.1 ± 11.4	93.0 ± 9.3	77.8 ± 13.5*†‡	85.2 ± 12	< 0.001
Heart rate (bpm)	66.4 ± 11.7	65.5 ± 11.3	73.8 ± 9.5	73.6 ± 16.0*	67.6 ± 12.8	< 0.001
QRS length (ms)	95.9 ± 11.8	100.1 ± 14.6*	100.2 ± 11.0	100.6 ± 15.4*	97.4 ± 13.1	0.001
Beta Blockers	90(23.1%)	26(28%)	3(37.5%)	17(16.7%)	136(22.9%)	0.208
ACEI / ARB	86(22.1%)	31(33.3%)	3(37.5%)	42(41.2%)*	162(27.3%)	< 0.001
Statins	156(40%)	54(58.1%)*	6(75.0%)	29(28.4%)†	245(41.3%)	< 0.001

*; significant difference towards No-CAD, †; difference towards PCI, ‡; difference towards CABG with *p* < 0.05

ACEI: Angiotensin Converting Enzyme Inhibitor, ARB; Angiotensin Receptor Blockers, BMI: Body mass index, CABG: Coronary artery bypass grafting, COPD: Chronic obstructive pulmonary disease, MI: Myocardial infarction, No-CAD: No coronary artery disease, PCI: Percutaneous coronary intervention

Table 2 displays the average segmental myocardial S/SR measurements with and without artefactual strain curves. Patients in the MI and the CABG groups had significantly reduced PLS and GLS than patients in the PCI and the No-CAD groups. In contrast, there were no significant differences in PLS and GLS measurements of the average segmental strain between the No-CAD and PCI groups. SRe was significantly reduced in the PCI group compared to the No-CAD group, was further reduced in the MI and CABG groups compared to the PCI group. SRs was significantly lower in the MI and CABG groups, and the prevalence of PSS was significantly higher in the MI group compared to the No-CAD group, whereas there were no significant differences of those measurements between the PCI and No-CAD groups. The S/SR values showed increased impairment for artefactual vs. non-artefactual strain curves in the No-CAD, PCI, MI groups, and not significantly in the CABG group. When comparing S/SR values with artefactual strain curves to those without artefacts, significant differences were found in SRe, SRs and PLS values in the No-CAD, PCI and MI groups, whereas these values were not significantly different in the CABG group. However, PSS and GLS showed no significant differences when comparing strain curves with and without artefacts across the four groups (Table 2).

**Table 2:** Myocardial strain and strain rate with and without artefact

	No-CAD	PCI	CABG	MI	*P* value
	*N* = 394	*N* = 94	*N* = 8	*N* = 102	
SRe (artefact-)	1.54 ± 0.27	1.43 ± 0.25^*^	1.12 ± 0.17^*†^	1.15 ± 0.36^*†^	< 0.001
SRe (artefact+)	1.51 ± 0.28^#^	1.38 ± 0.28^*#^	1.11 ± 0.17^*^	1.12 ± 0.35^*†#^	< 0.001
SRs (artefact-)	-1.18 ± 0.16	-1.15 ± 0.18	-1.11 ± 0.15	-0.98 ± 0.27^*^	< 0.001
SRs (artefact+)	-1.15 ± 0.16^#^	-1.12 ± 0.18^#^	-1.01 ± 0.1	-0.96 ± 0.26^*#^	< 0.001
PLS (artefact-)	-19.58 ± 2.18	-18.94 ± 2.27	-16.63 ± 2.79^*†^	-14.12 ± 4.69^*†^	< 0.001
PLS (artefact+)	-18.81 ± 2.4^#^	-18.04 ± 2.84^#^	-14.71 ± 2.51^*†^	-13.66 ± 4.56^*†#^	< 0.001
PSS (artefact-)	-0.70 ± 0.48	-0.70 ± 0.44	1.06 ± 1.13	-1.55 ± 1.03^*^	< 0.001
PSS (artefact+)	-0,71 ± 0,47	-0,70 ± 0,43	-1,09 ± 1.04	-1,51 ± 1,02^*†^	< 0.001
GLS (artefact-)	-18.81 ± 2.4	-18.23 ± 2.5	-14.72 ± 2.68^*†^	-14.29 ± 4.08^*†^	< 0.001
GLS (artefact+)	-18.73 ± 2.4	-17.95 ± 2.79	-14.77 ± 2.14^*†^	-14.28 ± 4.07^*†^	< 0.001

*; significant difference towards No-CAD, †; difference towards PCI, ‡; difference towards CABG with *p* < 0.05

#; significant difference towards artefact- with *p* < 0.05

CABG: Coronary artery bypass graft, CAD: Coronary artery disease, GLS: Global longitudinal strain, MI: Myocardial infarction, PCI: Percutaneous coronary intervention, PLS: segmental peak longitudinal strain during systole, PSS: Post systolic shortening, SRe: peak early diastolic strain rate, SRs: Peak systolic strain rate

**Fig. 1:**
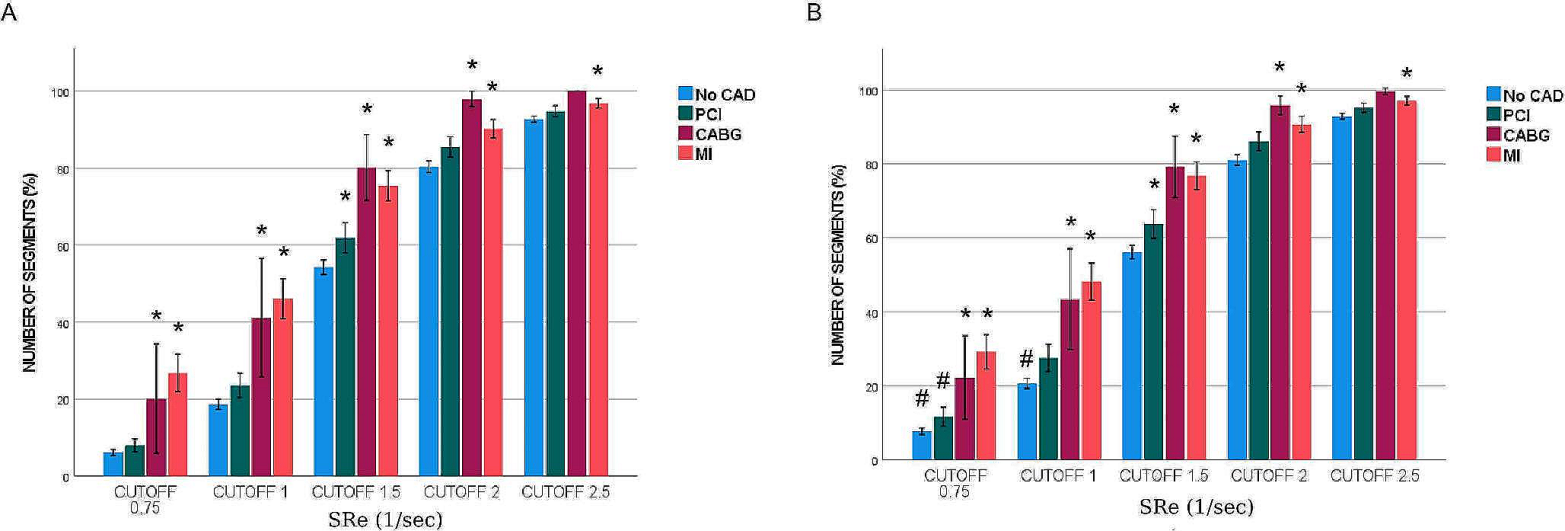
Comparing percentage of pathological segments of SRe (1/sec) across four groups, without (**A**) and with (**B**) artefacts *; significant difference towards No-CAD, #; significant difference towards artefact with *p* < 0.05

**Fig. 2:**
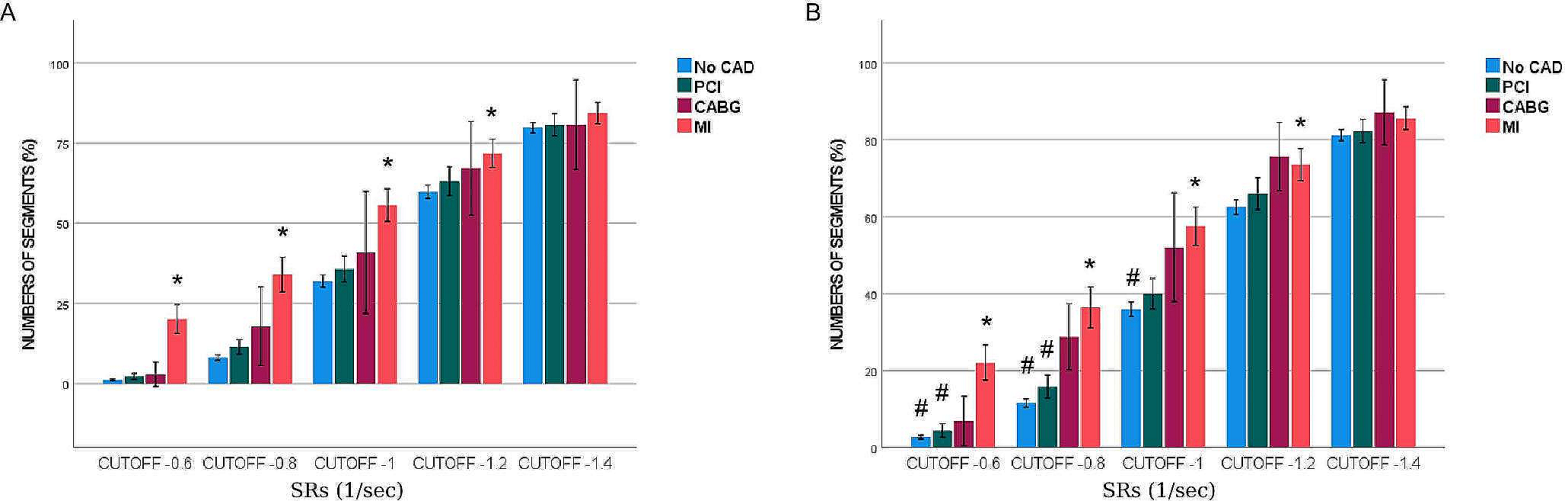
Comparing percentage of pathological segments of SRs (1/sec) across four groups, without (**A**) and with (**B**) artefacts *; significant difference towards No-CAD, #; significant difference towards artefact with *p* < 0.05

Figure 1 displays the percentage of pathological segments at different threshold values for reduced function (cut-off) without (Fig. 1A) and with (Fig. 1B) curve artefacts. For SRe, the percentage of pathological segments in the MI group were significantly higher compared with the No-CAD group at all cut-off values. Similarly, the number of pathological segments was significantly higher in the CABG group compared to the No-CAD group at cut-off values of 2.0 s^− 1^, 1.5 s^− 1^, 1.0 s^− 1^, 0.75 s^− 1^. On the other hand, number of pathological segments differed significantly between the PCI and No-CAD groups at the cut-off value of 1.5 s^− 1^ (Fig. 1). Analyses of pathological segments using various SRs cut-off values showed that the MI group had significantly higher number of pathological segments compared with the No-CAD group at all cut-off values without (Fig. 2A) and with (Fig. 2B) curve artefacts. However, there were no statistically significant differences in number of pathological segments when the CABG and PCI groups compared to the No-CAD group (Fig. 2).

**Fig. 3:**
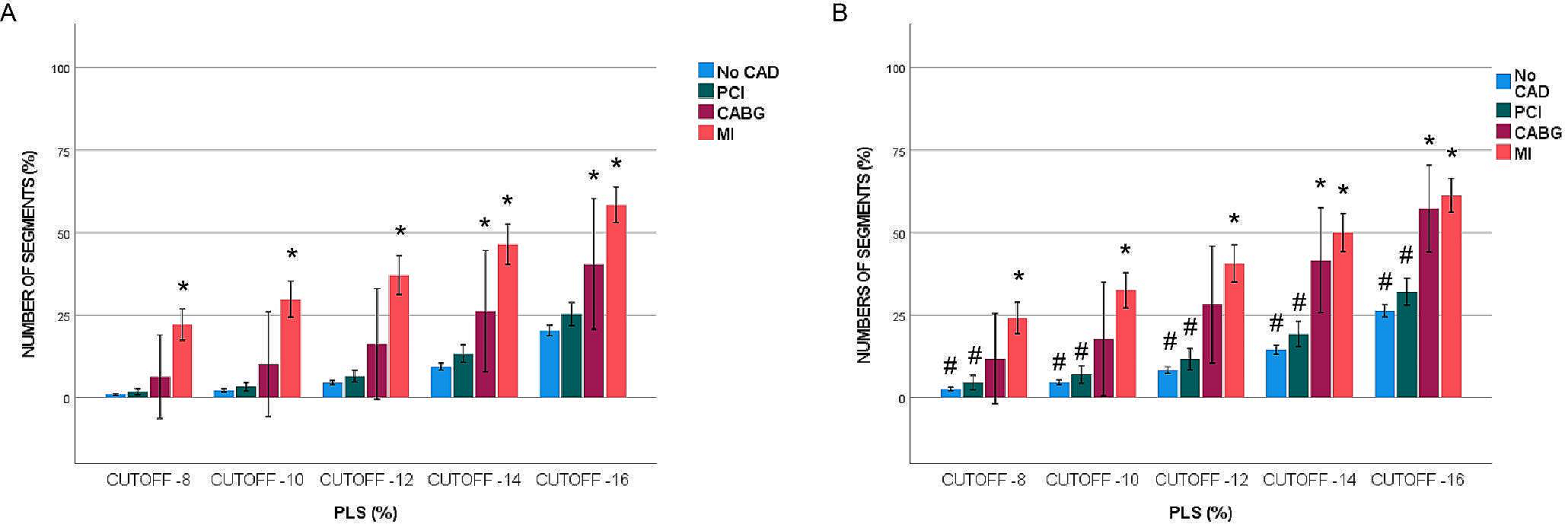
Comparing percentage of pathological segments of PLS (%) across four groups, without (**A**) and with (**B**) artefacts *; significant difference towards No-CAD, #; significant difference towards artefact with *p* < 0.05

**Fig. 4:**
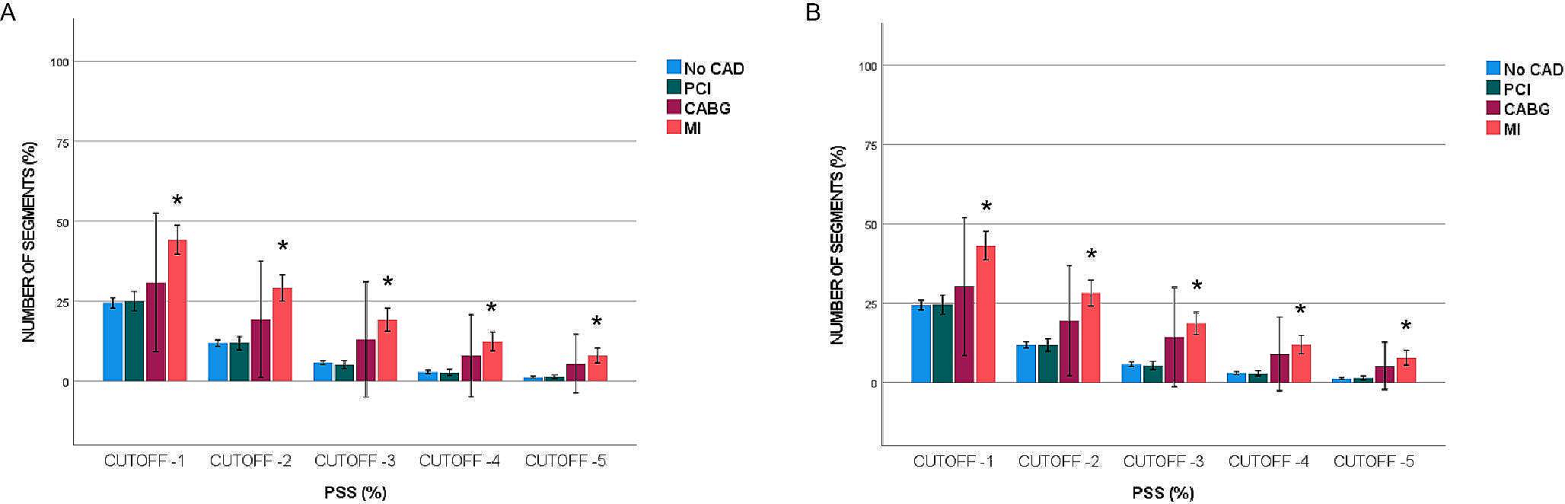
Comparing percentage of pathological segments of PSS (%) across four groups, without (**A**) and with (**B**) artefacts *; significant difference towards No-CAD, #; significant difference towards artefact with *p* < 0.05

Percentage of pathological segments at cut-off values of -16% and − 14% was significantly higher in the MI and CABG groups compared with the No-CAD group, while there were no significant differences in the percentage of pathological segments between the PCI and No-CAD groups. The percentage of pathological segments in the MI group was similar to the CABG group at cut-off values of -16%, and − 14%, whereas at cut-off values − 12%, -10%, and − 8%, the MI group had a significantly higher percentage of pathological segments than the PCI and CABG groups (Fig. 3). Regarding PSS, the MI group showed a significantly higher percentage of pathological segments at all cut-off values compared to the No-CAD group without (Fig. 4A) and with (Fig. 4B) curve artefacts. However, comparison of pathological segments using five different cut-off values for PSS showed no significant differences among the CABG, PCI and No-CAD groups (Fig. 4).

Table 3 demonstrates global endocardial, myocardial, and epicardial peak longitudinal strain (PLS) in these four groups. As expected, PLS increased from endocardial to epicardial in all groups. In line with other S/SR values, all PLS values indicated significant abnormalities in the CABG and MI groups compared with the No-CAD group. Although PLS was altered in the PCI group compared with the No-CAD group, the difference was not statistically significant.

**Table 3:** Segmental longitudinal endocardial, myocardial and epicardial strain in different groups

	No-CAD	PCI	CABG	MI	*P* value
	*N* = 394	*N* = 94	*N* = 8	*N* = 102	
PLS_endo	-22.97 ± 2.6	-22.18 ± 2.8	-20.14 ± 3.9*†	-21.74 ± 4.14*†	< 0.001
PLS_myo	-19.39 ± 2.2	-18.68 ± 2.8	-16.49 ± 2.8*†	-13.86 ± 4.8*†	< 0.001
PLS_epi	-16.81 ± 1.9	-16.20 ± 1.9	-13.99 ± 2.1*†	-11.89 ± 3.05*†	< 0.001

*; significant difference towards No-CAD, †; difference towards PCI, ‡; difference towards CABG with *p* < 0.05

PLS: segmental peak longitudinal strain during systole; endo: subendocardial; myo: midmyocardial; epi: subepicardial.

## Discussion

This study primarily investigated differences in resting S/SR values in a patient population with chronic chest pain by differentiating the patients with non-stenotic vs. stenotic coronary arteries. Revascularized MI patients were recruited as a pathological reference. Our findings provide several insights into the diagnostic value of segmental S/SR.

1. All average segmental S/SR parameters (SRe, SRs, PLS, GLS, PSS) differed significantly between the No-CAD and MI groups.
2. When comparing the percentage of segments with reduced function between the No-CAD and PCI groups, SRe showed a significant difference at a cut-off value of 1.5 s^-1^.
3. The percentage of pathological segments of SRe, PLS and GLS was significantly higher in the CABG group compared to the No-CAD group.
4. Our study also addressed strain curve artefacts, which can reduce segmental S/SR values. Notably, the percentage of pathological SRe, SRs and PLS segments was higher in analyses with compared to without artefacts in the No-CAD, PCI and MI groups. However, the presence of artefacts did not appear to disguise differences between groups to a clinically relevant degree.

The diagnosis of CAD using non-invasive imaging remains challenging. Patients with suspected stable CAD undergo various tests before referral to invasive CAG. A timely and accurate diagnosis of CAD could help many patients by enabling early treatment. Prior studies have focused on GLS analyses due to feasibility. However, GLS alone lacks sensitivity and specificity for diagnosing CAD, given its lack of detailed segmental analysis. Our study provides a new approach by grading segmental dysfunction through pathological segment counts. This detailed localized assessment of myocardial function may increase diagnostic accuracy compared to GLS.

### Percutaneous coronary intervention

Numerous studies have linked GLS to CAD severity [7–10, 17–19]. However, the heart is comprised of unique regions and layers. Specific myocardial segments may show distinct vulnerabilities requiring segmented analysis. Supporting this, prior work on layer strain found epicardial and mid-myocardial GLS predicted CAD [20]. In our study, endo- and epicardial strains were minimally different between the groups despite the endocardium being a more susceptible region to ischemia. The variations in myocardial strain among these groups can be attributed to the precision of the measurement technique, which is most reliable when applied across the entirety of myocardium. This discrepancy arises due to myocardium functions as a transmural unit and does not distinguish between endo- or epicardial dysfunction. Even though, in many cases, it is specifically subendocardial myocardium that sustains injury. Furthermore, GLS did not indicate CAD in PCI patients in our study. Similar to Hagemann et al., we enrolled patients with clinically suspected stable angina. Coexisting conditions like hypertension and diabetes may obscure S/SR differences between the groups. By stratifying patients and including the MI group as a reference, our results suggest GLS has limited sensitivity for low-risk CAD. Detailed segmental S/SR better detected subtle dysfunction.

As recurrent ischemia can impair relaxation prior to overt dysfunction, SRe may highlight differences between the No-CAD and PCI groups. Previous studies identified reduced SRe in CAD patients [4, 5], and improved diagnosis of CAD by adding SRe to exercise ECG [4]. Our findings showed that SRe differentiated the No-CAD group from CAD patients. Moreover, previous studies found that SRe and the ratio of peak mitral inflow velocity during early diastole compared to global early diastolic strain rate (E/SRe) independently predicted major adverse outcomes like MI, HF, AF and stroke after CAD treatment [4]. An additional study revealed that myocardial S/SR decreased as the severity of CAD increased. In predicting CAD severity, the study reported SRs and SRe outperformed longitudinal segmental strain [12]. In line with these findings, our results demonstrated that SRe with a cut-off value of 1.5 s^− 1^ was the most sensitive S/SR parameter to differentiate patients without CAD from patients with any type of CAD.

### Coronary artery bypass graft

Severe chronic CAD often impairs myocardial function, reflecting extensive damage due to the disease process. Accordingly, CABG patients are at higher risk for cardiovascular morbidity and mortality. Patients undergoing CABG have typically severe CAD and impaired myocardial function [21]. This impairment may be reflected by reduced segmental S/SR. In our study, CABG patients exhibited significantly decreased SRe, GLS and PLS. Although the number of CABG patients was low in the present study, segmental function in most of the CABG patients was highly impaired, enabling a high detection rate of high risk CAD patients.

Previous studies used S/SR values to evaluate left ventricle function after treating CAD with CABG [11, 22]. Resting segmental strain effectively identified viable myocardium and functional improvement post-CABG [22]. Additionally, a recent study found that the E/SRe ratio independently predicted all-cause mortality after CABG [11]. In this study, pathologically high E/SRe ratio was strongly associated with mortality in CABG patients with preserved left ventricular ejection fraction and GLS [11]. In line with these results, our study suggests that patients with pathologically low SRe might need closer follow-up to prevent adverse events [11].

Our findings indicate substantial global and regional myocardial dysfunction in the CABG group. By linking advanced CAD to abnormal S/SR values, our study highlights the potential utility of S/SR analyses for diagnosing severe and thus high risk-CAD and assessing the extent of myocardial dysfunction prior to CABG.

### Revascularized myocardial infarction

We recruited MI patients as a reference group to compare pathological segments of stable CAD with expected pathology of MI segments. Prior studies investigating utility of echocardiography after MI focused on prognostic value of strain rate measurements. For instance, Ersboll et al. demonstrated that E/SRe was independently associated with adverse outcomes after acute MI. E/SRe also indicated myocardial relaxation impairment better than velocity based analysis [15]. This study examined patients before and after CAG. In contrast, we analysed S/SR to investigate the effect of stunning on myocardium. Thus, we only included MI patients after revascularization.

Other studies have found reduced SRs and SRe in transmural infarcted segments and non-viable myocardium [14, 16]. Despite not differentiating transmurality or viability, we found significantly decreased SRe and SRs in the MI group compared to the No-CAD group. Furthermore, prior work has shown PSS and reduced SRs in ischemic vs. non-ischemic segments of suspected CAD patients [23]. Aligning with this, MI patients in our study had significantly lower SRs and higher PSS compared to the No-CAD group. Thus, our study revealed that several S/SR parameters, including GLS, PSS, PLS, SRe, and SRs, were significantly reduced in acute MI 1–3 days after revascularization. This indicates stunned myocardium exhibits persistent abnormalities in the first days after revascularization.

### Influence of strain curve-artefacts

Prior work of our group indicates strain curve artefacts are associated with reduced S/SR, potentially compromising the interpretation [13]. However, whether artefacts mask the strain rate differences among different patient groups was unclear. Our study found significant differences in SRe, SRs and PLS values when comparing strain curves with and without artefacts in the No-CAD, PCI and MI groups. This suggests artefacts may further reduce S/SR. Yet, despite the significant disparity between analyses with and without artefacts, the discrepancies among the No-CAD, PCI and MI groups remain relatively consistent. Hence, the presence of artefacts did not appear to obscure clinically relevant divergences. Interestingly, the CABG group showed no S/SR differences between strain curves with and without artefacts, potentially attributable to the small sample size (*n* = 8). Additionally, PSS and GLS did not significantly differ between artefact-present and artefact-free curves. This may indicate that the impact of artefacts may vary among different strain parameters. In summary, while artefacts influenced measured S/SR values, the relative differences between patient groups remained intact, suggesting artefact presence did not substantially disguise the underlying relationships.

### Clinical implications

Our results have several clinical implications. Firstly, the study supports utility of echocardiography as a non-invasive and supplementary modality to diagnose CAD. Secondly, detailed S/SR analysis rather than GLS only measurements may improve diagnostic accuracy in patients with milder pathology, where GLS lacks specificity. Thirdly, SRe could aid in diagnosing patients with chronic chest pain who need referral to invasive CAG. Finally, differential segmental strain patterns could indicate localized pathological changes underlying CAD.

### Limitations of the study

While the study included a large cohort of patients, the sample size in the CABG group was relatively small. While this limitation might prevent obtaining statistically significant differences in the CABG group compared with the other groups in certain comparisons, the trend of differences across the groups are visible and indicate validity of comparisons. However, larger groups with similar numbers of participants could provide more robust comparisons. Evaluating appropriate reference groups for stable CAD poses challenges. We used acute MI patients after revascularization. When compared to myocardial dysfunction in patients with chronic CAD, the patients in the MI group might have unrelated changes in myocardial function due to revascularization damage and oedema.

## Conclusion

In summary, our study demonstrates the potential of detailed segmental strain analysis to identify myocardial dysfunction consistent with CAD severity. Patients who had revascularized MI or needed CABG had significantly reduced segmental S/SR values compared to the No-CAD group. For chronic chest pain, SRe indicated differences between the No-CAD and PCI groups. Further research on segmental S/SR in stable CAD and diagnostic utility of SRe is warranted.

## Electronic supplementary material

Below is the link to the electronic supplementary material.

Supplementary Material 1

## Data Availability

No datasets were generated or analysed during the current study.

## Abbreviations

AF: Atrial fibrillation
CABG: Coronary artery bypass graft
CA: Coronary artery
CAD: Coronary artery disease
CAG: Coronary angiography
CCTA: Coronary computed tomography angiography
COPD: Chronic obstructive pulmonary disease
DM: Diabetes mellitus
GLS: Global longitudinal strain
HF: Heart failure
HT: Hypertension
LBBB: Left bundle branch block
LVOT: Left ventricular outflow tract
MI: Myocardial infarction
PCI: Percutaneous coronary intervention
PLS: Peak longitudinal strain during systole
SRs: Peak systolic strain rate
SRe: Peak early diastolic strain rate
S/SR: Strain and strain rate
2DSTE: Two-dimensional speckle tracking echocardiography
