# Supplementary material for: Resting segmental speckle tracking strain and strain rate in stable coronary artery disease and revascularized myocardial infarction
Source: Int J Cardiovasc Imaging. 2024 Aug 23;40(10):2077–86. doi: 10.1007/s10554-024-03200-0 (PMC11499336; doi:10.1007/s10554-024-03200-0)
Supplement: Supplementary file 1 — Supplementary Material 1 [file 10554_2024_3200_MOESM1_ESM.docx]

**Supplementary information**

*Assessment of intra-observer and inter-observer variation in S/SR measurements*

Supplementary figure 1 shows intra-observer variation of PLS, SRe, and SRs. The mean difference ± 1.96 SDs was 0.07±0.3 s^-1^ for SRe, -0.03 ± 0.22 s^-1^ for SRs, and -0.88 ± 2.83 s^-1^ for PLS. Supplementary figure S2 shows inter-observer variation of PLS, SRe, and SRs. The mean difference ± 1.96 SDs was -0.07±0.4 s^-1^ for SRe, -0.04± 0.27 s^-1^ for SRs, and -0.1 ± 4.4% for PLS.


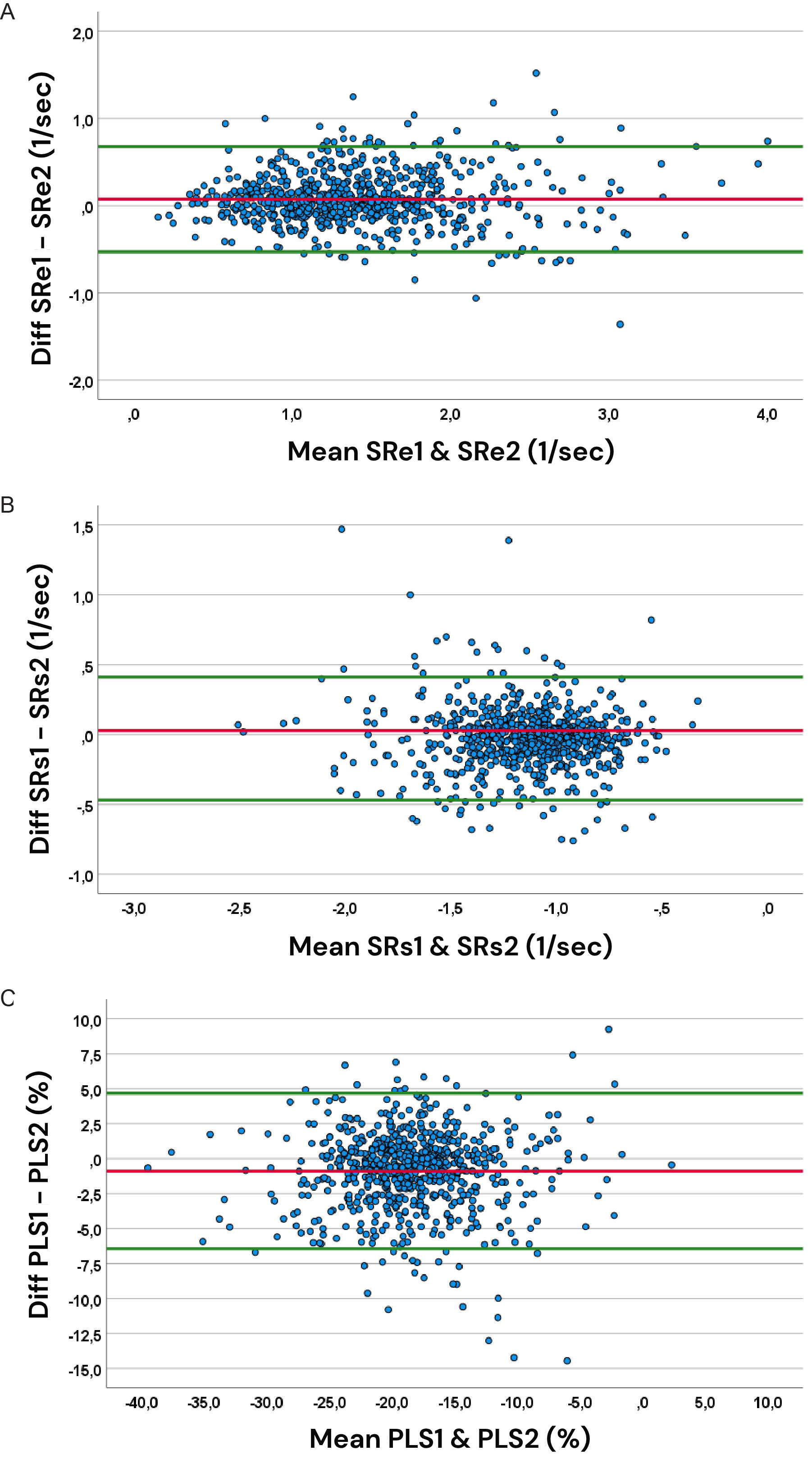


Supplementary figure 1. Bland-Altman plot for intra-observer variation analysis of SRe (1/sec), SRs (1/sec) and PLS (%).


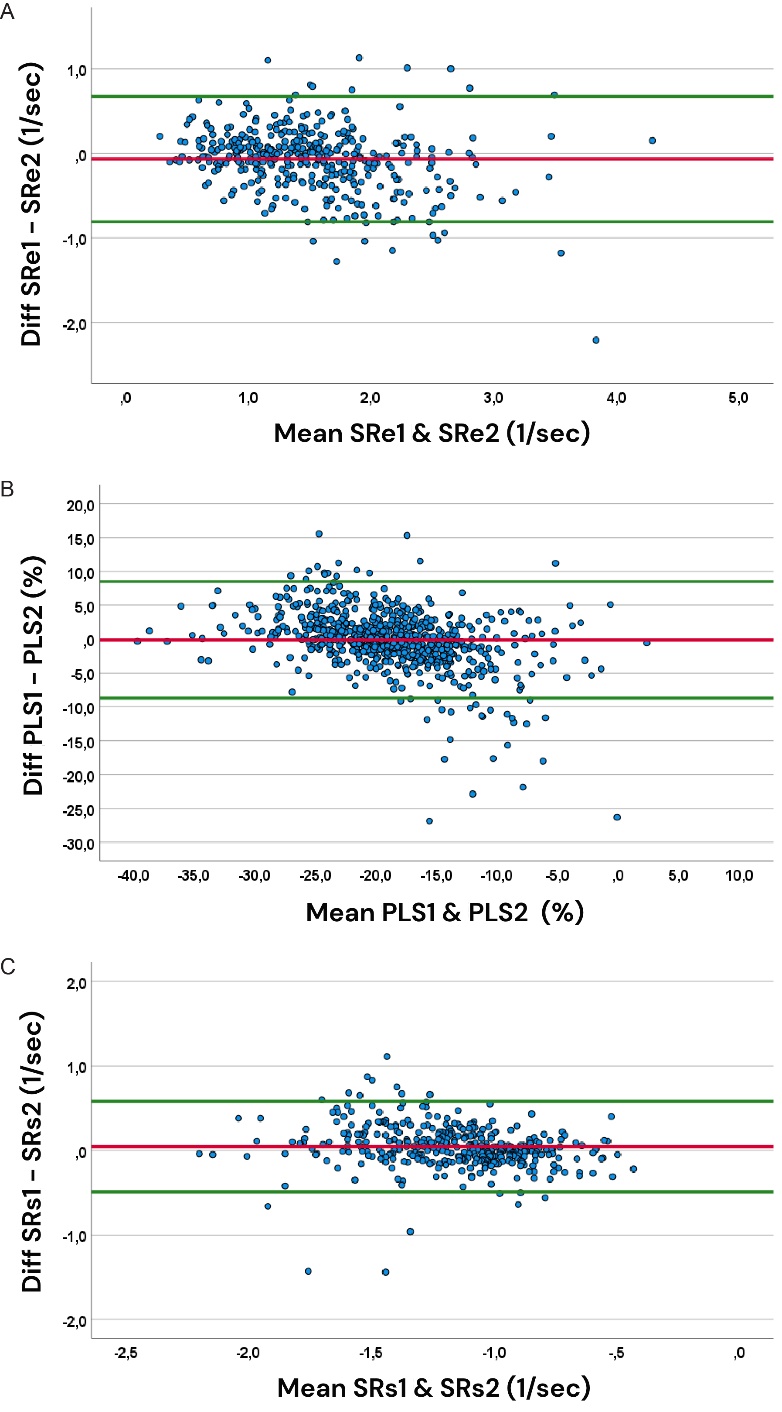


Supplementary figure 2. Bland-Altman plot for inter-observer variation analysis of SRe (1/sec), SRs (1/sec) and PLS (%).
